# Reassessment of the role of CaCO_3_ in n-butanol production from pretreated lignocellulosic biomass by *Clostridium acetobutylicum*

**DOI:** 10.1038/s41598-020-74899-9

**Published:** 2020-10-21

**Authors:** Zengping Su, Fengqin Wang, Yaohuan Xie, Hui Xie, Guotao Mao, Hongsen Zhang, Andong Song, Zhanying Zhang

**Affiliations:** 1 Key Laboratory of Agricultural Microbial Enzyme Engineering (Ministry of Agriculture), College of Life Science, Henan Agricultural University, No. 63, Nongye Road, Jinshui District, Zhengzhou, 450002 Henan Province China; 2 Centre for Agriculture and the Bioeconomy, Institute for Future Environments, Queensland University of Technology, Brisbane, QLD 4000 Australia; 3 School of Mechanical, Medical and Process Engineering, Science and Engineering Faculty, Queensland University of Technology, Brisbane, QLD 4000 Australia

**Keywords:** Applied microbiology, Industrial microbiology

## Abstract

In this study, the role of CaCO_3_ in n-butanol production was further investigated using corn straw hydrolysate (CSH) media by *Clostridium acetobutylicum* CICC 8016. CaCO_3_ addition stimulated sugars utilization and butanol production. Further study showed that calcium salts addition to CSH media led to the increase in Ca^2+^ concentration both intracellularly and extracellularly. Interestingly, without calcium salts addition, intracellular Ca^2+^ concentration in the synthetic P2 medium was much higher than that in the CSH medium despite the lower extracellular Ca^2+^ concentrations in the P2 medium. These results indicated that without additional calcium salts, Ca^2+^ uptake by *C. acetobutylicum* CICC 8016 in the CSH medium may be inhibited by non-sugar biomass degradation compounds, such as furans, phenolics and organic acids. Comparative proteomics analysis results showed that most enzymes involved in glycolysis, redox balance and amino acids metabolism were up-regulated with CaCO_3_ addition. This study provides further insights into the role of CaCO_3_ in n-butanol production using real biomass hydrolysate.

## Introduction

Lignocellulosic biomass is the most abundant bioresource on Earth for producing renewable biofuels^1^. Although cellulosic ethanol is the most studied biofuel, n-butanol is more attractive because it has superior fuel properties, e.g., a relatively high energy density, a low water solubility, almost similar characteristics to gasoline, and compatible with current engines^2^. Butanol is also a versatile chemical. It is a chemical intermediate to produce butyl acrylate, methacrylate and plastics^2^. It is also used as a solvent in the production of glycol ethers and butyl acetate for manufacturing of paint, dyes and others^2,3^. In addition, n-butanol from fermentation processes can be used as an artificial flavor for food and beverage applications^3^.

The acetone–butanol–ethanol (ABE) fermentation process using *Clostridium* strains is the most promising bioprocess for butanol production. Butanol production from lignocellulosic biomass has gained increasing interests because the feedstock is abundant and low-cost and sugars-derived from lignocellulosic biomass, such as glucose, xylose, arabinose and mannose can be utilized by butanol producing clostridia strains^4–6^. However, due to the recalcitrant nature of lignocellulose, pretreatment is required to deconstruct the biomass in order to improve the sugars yield in the subsequent enzymatic hydrolysis step^2,7^. Pretreatment also leads to the generation of non-sugar biomass degradation compounds, which may have inhibitory effects on ABE fermentation^2,4,7^. These potential components include furans [furfural and 5-hydroxylmethylfurfural (HMF)], organic acids (formic acid, acetic acid, etc.) and soluble phenolic compounds^8,9^. In order to eliminate or reduce the inhibitory effects, detoxification of pretreatment hydrolysate with various approaches, such as glycerol supplementation and adsorption by activated carbon, resins and overliming has been applied to improve biobutanol production^4,7,10–13^.

Overliming with Ca(OH)_2_ or CaO is one of the mostly used methods to detoxify biomass hydrolysate derived from dilute acid or hot water pretreatments prior to butanol production^4,7,11^. A typical overliming process consists of a pH increase step (pH 10 adjusted by lime), an incubation step (60 °C for 2–6 h), a pH decrease step (pH 6–7 adjusted by H_2_SO_4_ for fermentation) and a filtration or centrifugation step to remove precipitates^14,15^. Overliming could effectively remove furans and many soluble phenolic compounds and alleviate the inhibitory effect on fermentation^14,15^. In addition, CaCO_3_ is often used as a neutralization and buffering agent for pH adjustment during butanol production^16–19^. Studies found that in addition to its role in pH adjustment, Ca^2+^ from CaCO_3_ also played roles in relieving carbon catabolite repression caused by the use of mixed carbon sources^20,21^, relieving butanol toxicity and ameliorating the inhibitory effects from non-sugar biomass degradation products^22^. In order to elucidate the role of CaCO_3_/Ca^2+^ and the inhibitory effect of non-sugar biomass degradation compounds, genetic, metabolic and proteomic analyses have been conducted^19,20,23–25^. However, most of these studies used CaCO_3_ or Ca^2+^-containing synthetic media^20,23,24^ or sole inhibitors such as furfural, formic acid and ferulic acid^19,25^. Comprehensive studies on the responses of *Clostridium* strains in the Ca^2+^-containing real biomass hydrolysate, especially at molecular levels, are still very limited. Liu et al. found that overliming removed the growth inhibitory effect of corn stover hydrolysate but was unable to eliminate the effects of soluble lignin compounds (SLC) on redox balance in *C. beijerinckii*, leading to a high acetone/butanol ratio^18^. It was also reported that thioredoxin reductase gene, *trxB*, was up-regulated in the late exponential phase, possibly to response to the increased oxidative stress caused by SLC^18^.

In this study, corn straw hydrolysate (CSH) derived from dilute acid pretreatment and enzymatic hydrolysis was used directly for ABE production by *Clostridium acetobutylicum* CICC 8016. ABE production was compared using two CSH media containing calcium salts (CaCO_3_ and CaCl_2_, respectively), one calcium salt-free CSH medium and one calcium salt-free synthetic P2 medium. Furthermore, the effects of additional calcium salts on the intra- and extra-cellular Ca^2+^ concentrations were investigated with the CSH and the synthetic P2 media. In addition, comparative proteomic analysis was undertaken to understand the responses of proteins with the CSH media in the presence of CaCO_3_. Based on the results, the role of CaCO_3_ in ABE fermentation of lignocellulosic hydrolysate was further revealed and discussed. This study is also one of the few comprehensive studies investigating the role of CaCO_3_ in ABE production using pretreated lignocellulosic biomass.

## Results and discussion

### Inhibitors in fermentation media

Table 1 shows the compositions of sugars and inhibitors in the media at the beginning (0 h) and the end (72 h) of the fermentation with and without the addition of CaCO_3_ and CaCl_2_, respectively. At the beginning of the fermentation, all the three media contained similar levels of sugars, HMF and soluble phenolic compounds. The concentration of furfural in control medium was higher than that in the media with calcium salts while the concentration of formic acid was lower. Changes in furfural and formic acid were possibly due to the hydrolysis of furfural to formic acid during sterilization in the presence of calcium salts. It was unclear why the concentration of acetic acid in the medium with the addition of CaCO_3_ was reduced. The concentrations of single inhibitors in the CSH media were lower than those reported levels causing inhibition on cell growth and ABE production. For example, previous studies found that *Clostridium* strains could tolerate up to at least ~ 1.0 g/L furfural, ~ 1.0 g/L HMF, and 0.2 g/L single phenolic compounds such as ferulic acid, syringaldehyde and *p*-courmaric acid without negative effect on cell growth and ABE production^8,26^. The low inhibitors concentrations were possibly due to the use of less severe pretreatment conditions and different feedstocks^22,27,28^. In this study, the CSH medium contained relatively high concentrations of acetic acid and formic acid but very low concentrations of furans and phenolic compounds (Table 1). These compounds may also have an amplified inhibition on cell growth and ABE production due to a synergistic effect.

**Table 1:** Composition change of CSH media following processing and treatment.

Fermentation time	Calcium salt	Glucose (g/L)	Xylose (g/L)	HMF (mg/L)	Furfural (mg/L)	HBA (mg/L)	Vanillin (mg/L)	Coumaric acid (mg/L)	Ferulic acid (mg/L)	Formic acid (mg/L)	Acetic acid (g/L)
0 h	Control (no addition)	15.7 ± 0.3	11.8 ± 0.7	18.2 ± 2.1	12.4 ± 0.3	2.5 ± 0.0	5.8 ± 0.1	61.8 ± 1.7	27.6 ± 0.4	648.8 ± 8.6	3.4 ± 0.4
	CaCO_3_	14.4 ± 1.5	11.4 ± 0.8	18.1 ± 1.0	9.9 ± 0.4	2.5 ± 0.1	5.4 ± 0.4	61.6 ± 3.6	26.0 ± 1.7	734.7 ± 11.6	1.9 ± 0.2
	CaCl_2_	15.1 ± 0.5	11.4 ± 0.7	18.0 ± 0.5	9.5 ± 0.3	2.7 ± 0.6	5.3 ± 0.2	61.2 ± 2.3	25.9 ± 0.3	712.6 ± 14.7	2.7 ± 0.2
72 h	Control (no addition)	4.2 ± 0.4	10.5 ± 0.9	0.4 ± 0.2	0.7 ± 0.0	0.0 ± 0.0	0.5 ± 0.1	0.0 ± 0.0	0.0 ± 0.0	594.5 ± 49.3	4.3 ± 0.5
	CaCO_3_	0.0 ± 0.0	0.0 ± 0.0	0.6 ± 0.2	0.0 ± 0.0	0.0 ± 0.0	0.0 ± 0.0	0.0 ± 0.0	0.0 ± 0.0	275.2 ± 3.6	5.4 ± 0.5
	CaCl_2_	1.6 ± 0.5	6.2 ± 0.1	0.5 ± 0.1	0.0 ± 0.0	0.0 ± 0.0	0.0 ± 0.0	0.0 ± 0.0	0.0 ± 0.0	319.3 ± 70.3	5.1 ± 0.3

*HMF* 5-hydroxymethylfurfural, *HBA* 4-hydroxybenzaldehyde.

At the end of the fermentation, except for acetic acid all the other biomass degradation compounds concentrations decreased significantly or to undetectable levels, especially with calcium salts. The concentration reduction of biomass degradation compounds was possibly due to consumption by the strain. Studies have shown *Clostridium* strains were able to metabolize furans such as HMF and furfural to their alcohols and phenolics such as coumaric acid to phloretic acid with reduced toxicity^25,29^. Indeed, low concentrations of furfural (no more than 1 g/L) and HMF (no more than 2.0 g/L) improved ABE production^8,30^. The improved ABE production was attributed to enhanced NAD^+^ regeneration, which accelerated the oxidative step of the glycolytic pathway and ultimately increasing glycolysis with furan eventually being transformed to their less toxic alcohols^30^. With regard to carboxylic acids, acetic acid itself is an intermediate in ABE production. *C. acetobutylicum* ATCC 824 and *C. beijerinckii* NCIMB 8052 could tolerate high concentration of acetic acid up to 9.7 g/L^31^. However, formic acid is much more toxic to clostridia strains. One study showed that formic acid concentration higher than 0.02 g/L and 0.04 g/L had negative impacts on butanol production and cell growth by *C. acetobutylicum*, respectively^26^. Formic acid up to 1 mM (0.046 g/L) could fail the transition from acidogenesis to solventogenesis, triggering “acid crash” by *C. acetobutylicum* in pH uncontrolled media^32^. Studies also showed that the tolerance of formic acid varied significantly and *C. beijerinckii* NCIMB 8052 could tolerate up to 1.0 g/L formic acid without negative effects on cell growth and ABE production^31^. This is because *C. beijerinckii* strains have formate dehydrogenase (FDH), which oxidizes formic acid to carbon dioxide^22,24^. In addition, formic acid might have also been converted to pyruvate and CoA by pyruvate formate lyase (PFL)^33^. In this study, the reduction of formic acid concentration by *C. acetobutylicum* CICC 8016 may be due to conversion by PFL or be reutilized as one-carbon unit donor^32^.

### Effect of exogenous Ca^2+^ on ABE fermentation in ***C. acetobutylicum*** CICC 8016

**Figure 1:**
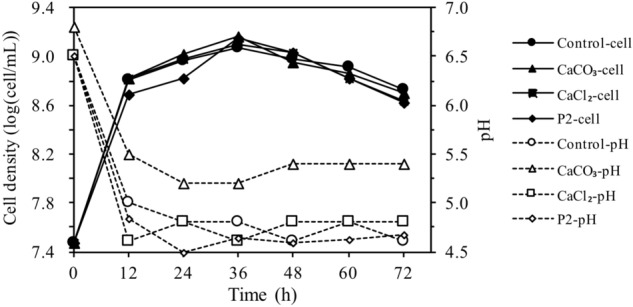
Cell density and pH during ABE production by *C. acetobutylicum* CICC 8016 with CSH and P2 media. 50 mM CaCO_3_ or CaCl_2_ was added to the CSH medium.

Figure 1 shows the cell densities and pH changes during ABE production using the hydrolysate media. It appeared that cultivation media (CSH medium and P2 medium) and media calcium ions (with or without Ca^2+^) had little effect on cell growth (Fig. 1). After 36 h, cell densities reduced with all media. pH values of all the four media dropped sharply in the first 12 h. After 12 h, pH values of the medium with CaCO_3_ maintained at a higher range of 5.2–5.4 than those of the other three media (pH 4.5–4.8) due to the buffering capacity of CaCO_3_ (Fig. 1). Cell densities in the CSH medium containing CaCO_3_ were also relatively high during 12–36 h compared to those in other media.

**Figure 2:**
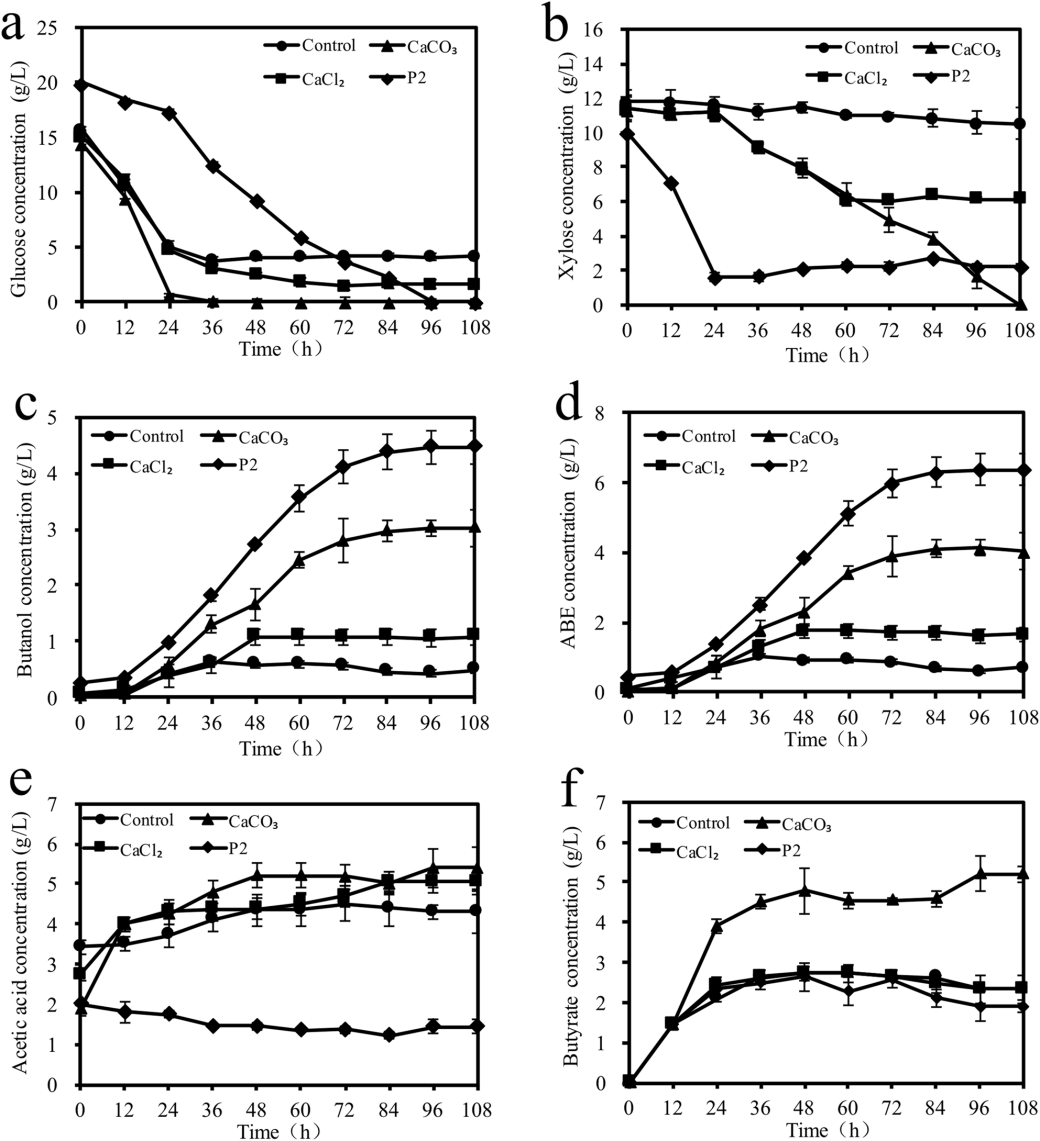
Sugar consumption, ABE and acid production with CSH and P2 media and P2 medium. 50 mM CaCO_3_ or CaCl_2_ was added to the CSH medium.

Figure 2 shows the effect of CaCO_3_ and CaCl_2_ on sugars consumption, ABE production and by-products accumulation in the CSH media. In the first 24 h, glucose consumption with the CSH control medium (no additional calcium salts) was more rapid than that with the inhibitors-free synthetic P2 medium though the pH changes in both media had similar trend. *C. acetobutylicum* CICC8016 used glucose and xylose simultaneously in the inhibitors-free synthetic P2 medium. However, with the CSH control medium, little xylose was consumed. These results indicated that micronutrients in CSH medium stimulated glucose consumption and non-sugar biomass degradation products inhibited xylose assimilation. With the addition of calcium salts, the use of xylose started from the depletion of glucose and/or with reduced glucose concentrations in the CSH media. With CaCl_2_, xylose consumption stopped after 60 h with a residual xylose concentration of ~ 6 g/L. With P2 medium, xylose consumption stopped after 24 h with a residual xylose concentration of ~ 2 g/L. The highest butanol and ABE concentrations were achieved with the P2 medium, followed by those with the CSH media containing CaCO_3_ and CaCl_2_, respectively, and those with the CSH control medium. Acetic acid concentrations in the three CSH media were much higher than those in the P2 medium. In contrast, butyric acid concentrations in the CaCO_3_-containing hydrolysate medium were much higher than those in all the other media after 12 h fermentation.

Many clostridia strains exhibit diauxic growth on two carbon sources. When growing on glucose and xylose media, these clostridia strain consume glucose firstly, followed by xylose utilization^6,34^. However, some clostridia strains, such as *Clostridium beijerinckii* strain BA101 and P260 were able to consume glucose and xylose simultaneously though glucose consumption was faster than xylose^9,35^. In this study, it was observed that xylose consumption stopped at 24 h with P2 medium and at 60 h with CaCl_2_ medium, which was possibly attributed to the low medium pH (Fig. 1) that inhibited the activities of enzymes related to xylose metabolism. However, xylose consumption stopped at different times indicated that xylose metabolism was affected by many catabolite regulation factors, including catabolite repression by glucose, and the effects from pH, biomass and calcium ions. These results also indicated that CaCO_3_ addition played multi-roles, including the pH buffering effect, regulating the balance of NADH and ATP by Ca^2+^ for solvent production, and alleviating the negative effect from biomass inhibitory compounds^17,19,22^, thus stimulating sugar utilization and butanol production by *Clostridium* species^20,21,36^.

Although CaCO_3_ addition could improve butanol and ABE production using CSH hydrolysate, butanol and ABE concentrations were still much lower than those with P2 medium, indicating that CaCO_3_ could not eliminate the inhibitory effect of using CSH hydrolysate completely. The high acetic acid concentrations in CSH media (even with CaCO_3_ addition) and high butyric acid concentrations in CaCO_3_-containing CSH medium compared to those with P2 medium indicated that the organic acids produced in the acidogenic phase could not be effectively converted to corresponding solvents. The less effective transformation from acidogenesis to solventogenesis was possibly due to the inhibitory effects of non-sugars biomass degradation compounds.

### Extracellular and intracellular Ca^2+^ concentrations during ABE fermentation

**Figure 3:**
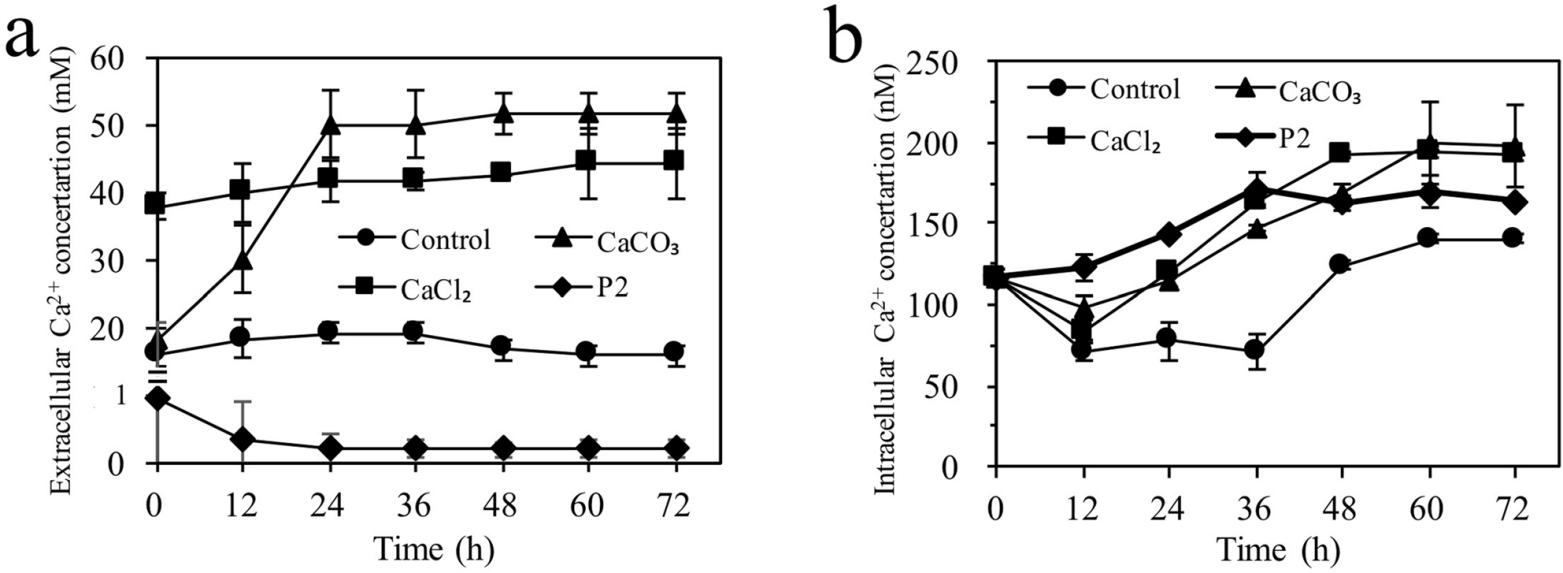
Extracellular (**A**) and intracellular (based on 1 × 10^5^ cells/mL) (**B**) Ca^2+^ concentrations in *C. acetobutylicum* cells during ABE fermentation with CSH media and P2 medium. 50 mM CaCO_3_ or CaCl_2_ was added to the CSH medium.

Figure 3 shows the extracellular and intracellular Ca^2+^ concentrations during ABE fermentation. Both the CSH control medium and P2 medium did not have additional calcium salts, but the levels of extracellular and intracellular Ca^2+^ concentrations were different. The extracellular Ca^2+^ concentrations in the P2 medium were the lowest, less than 1 mM, much lower than the second lowest Ca^2+^ concentrations of ~ 15–18 mM in the CSH control medium. However, the intracellular Ca^2+^ concentrations with the P2 medium were higher than those in the CSH control medium though the P2 medium had much lower extracellular Ca^2+^ concentrations and the two media had similar levels of pH values during fermentation. These results indicated that calcium uptake by *C. acetobutylicum* CICC 8016 in the CSH control medium was inhibited by the presence of the inhibitors. The lowest solvent production in the CSH control medium was likely attributed to the lowest intracellular Ca^2+^ concentrations (Fig. 2).

Addition of calcium salts to the CSH medium increased both extracellular and intracellular Ca^2+^ concentrations compared to the CSH control medium. Addition of CaCl_2_ had an immediate effect on the extracellular Ca^2+^ concentration at the beginning of the fermentation and the extracellular Ca^2+^ concentration remained relatively stable during the fermentation. Addition of CaCO_3_ increased the Ca^2+^ concentration gradually in the first 24 h as it took time to dissolve CaCO_3_. After 24 h, the extracellular Ca^2+^ concentrations in the CaCO_3_-containing medium surpassed those in the CaCl_2_-containing medium and remained stable till the end of fermentation.

The intracellular Ca^2+^ concentrations in the calcium salts-containing CSH media and the P2 medium increased gradually till reached to plateau with the prolonged fermentation time, perhaps being prepared for endogenetic spore formation^23^. In a previous study, Han et al. also investigated the effect of the addition of calcium salt (CaCl_2_ and CaCO_3_) on intracellular Ca^2+^ levels in *C. beijerinckii* using P2 media and observed 2–3 times increase in intracellular Ca^2+^ concentration compared to control without calcium salts^20^. They concluded that CaCO_3_ was not only a buffering agent but also a source of calcium ions to affect the intracellular level of Ca^2+^^20^. The role of exogenous calcium salts in increasing the intracellular Ca^2+^ concentration was also proved in this study.

Intracellular Ca^2+^ inhibitor BAPTA-AM is recognized as an international standard tool drug used in Ca^2+^ complexation. BAPTA binds intracellular free Ca^2+^, thus controlling intracellular Ca^2+^ levels^37,38^. In order to further investigate the role of intracellular Ca^2+^ on ABE fermentation by *C. acetobutylicum*, 20, 30 and 40 μM BAPTA-AM were added to the P2 media in different ways. As shown in supplementary data (see Supplementary Fig. S1 online) without BAPTA-AM addition, *C. acetobutylicum* consumed more glucose (though the effect on xylose consumption was unclear) and produced more ABE and butanol (though the effect on organic acids production was unclear). Han et al. used EDTA as a calcium ion chelator and found that addition of EDTA (1 mM) inhibited *C. beijerinckii* growth and solventgenesis^20^. Supplement of Fe^2+^, Mg^2+^ and Mn^2+^ (metal ions involved in glycolysis and ABE synthesis) was unable to alleviate this EDTA-induced inhibition while supplement of additional 0.5 g/L Ca^2+^ alleviated the inhibition on growth and solventogenesis. The results of our study with the addition of different Ca^2+^ chelator further proved the role of intracellular Ca^2+^ in ABE production. In addition, the results of our study indicated that in the CSH control medium, non-sugars biomass degradation products likely inhibited the uptake of calcium ions into the cells, leading to low levels of intracellular Ca^2+^ and poor ABE production.

### Comparative proteomics analysis of ***C. acetobutylicum*** by addition of CaCO_3_

It has been reported that Ca^2+^ addition led to significant level increase of proteins predominantly involved in heat shock response, DNA synthesis, transcription and repair, carbohydrate metabolism/transport and signal transduction of *C. beijerinckii* strains^20,24^. In addition, CaCO_3_ up-regulated amino acid metabolism was also reported for degenerated *C. beijerinckii* strain^23^. In this study, proteomics was performed to investigate the possible mechanism of Ca^2+^ addition for enhancing butanol production from CSH. A total of 281 protein spots were resolved, of which 54 proteins showed level differences and 30 proteins displayed level change of > 1.50-fold. Among the 30 proteins, levels of 25 proteins increased and the other 5 decreased (Table 2). The largest number of proteins was related to the metabolism of carbohydrate and energy (8 of 30 proteins). Five proteins associated with metabolism of amino acid. Ten proteins related to DNA replication, nucleotide metabolism, protein processing and glycolipid synthesis were also overexpressed in CSH medium with addition of CaCO_3_ (Table 2).

**Table 2:** Complete lists of significant protein level changes of *C. acetobutylicum* using CSH medium with CaCO_3_ addition.

Protein	UniProtKB accession	p*I*	Fold change
Pyruvate kinase	O08309	5.74	+ 22.34
Pyruvate flavodoxin/ferredoxin oxidoreductase	Q97GY6	5.85	+ 4.26
Deacethylase/dipeptidase/desuccinylase family of Zn-dependent hydrolases	Q97FL3	4.89	+ 9.98
2,3-bisphosphoglycerate-independent phosphoglycerate mutase	Q97L53	5.29	+ 9.98
Acetyl CoA acetyltransferase (Thiolase)	Q7DFN1	5.72	+ 23.08
dTDP-glucose 4,6-dehydratase	Q97GN4	5.50	+ 4.41
Phosphoribosylformylglycinamidine cyclo-ligase	Q97J93	5.22	+ 6.92
50S ribosomal protein L2	Q97EI1	5.14	+ 1.99
ATP-dependent Clp protease proteolytic subunit	P58276	5.03	+ 2.57
PLP-dependent aminotransferase	Q97FA8	5.37	+ 3.39
Pyruvate formate lyase	G7MD03	5.60	+ 5.93
DNA ligase D	G9XJI5	6.68	+ 5.93
Aminotransferase	A5Z9L3	4.91	+ 2.30
Monogalactosyldiacylglycerol synthase	D4KPI2	9.10	+ 2.30
Glycogen synthase	E9SG36	5.44	+ 1.93
Ferredoxin	P00216	5.77	+ 21.50
Iron-regulated ABC-type transporter membrane component (SufB)	Q97E27	5.32	+ 9.98
Methionine aminopeptidase	P69000	5.14	+ 4.67
Prophage antirepressor	D6DF48	9.04	+ 5.93
Mismatch repair ATPase (MutS family)	R7L2G8	5.11	+ 1.93
Helicase C-terminal domain protein	J5UCQ1	4.99	+ 21.50
NADH-dependent flavine oxidoreductase	Q97MK0	5.30	+ 1.93
Aminotransferase	B9E028	4.91	+ 4.12
Hypothetical protein	R9IJU6	9.51	+ 21.50
ATP-binding protein	R5TY26	6.20	+ 3.87
M18 family aminopeptidase	D8GJT7	5.25	− 1.80
DNA topoisomerase	T4NJI5	7.04	− 1.54
Hypothetical protein	Q97MY3	4.67	− 50.00
Protein containing cell adhesion domain	Q97EM4	4.77	− 84.89
Ribulose-phosphate 3-epimerase	Q97IC0	5.76	–^a^

^a^Not detected after CaCO_3_ addition.

**Figure 4:**
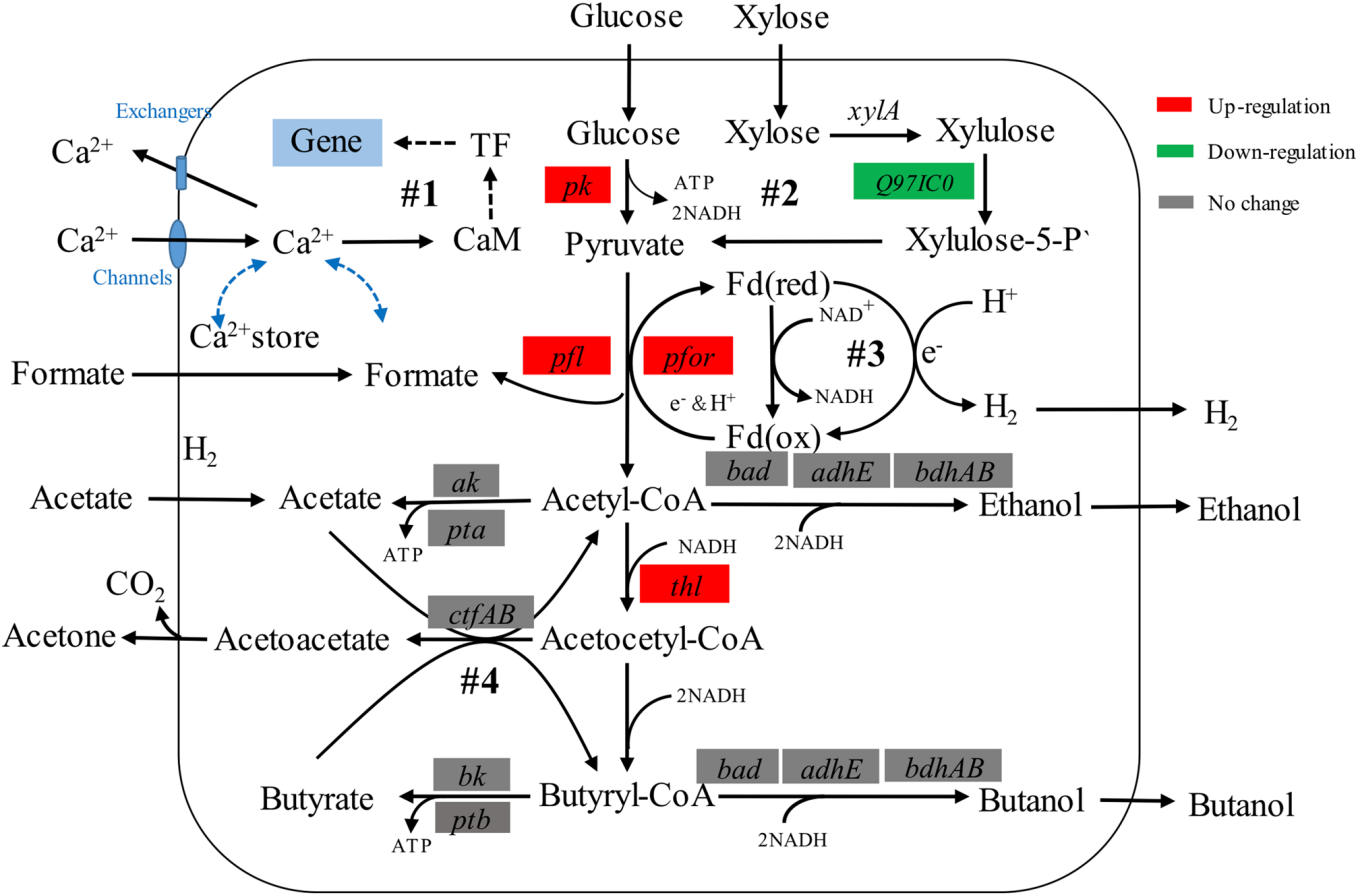
The *C. acetobutylicum* metabolism map with 50 mM CaCO_3_ addition.

Based on proteomics analysis results, Fig. 4 summarized the effect of CaCO_3_ addition on the metabolic pathway of *C. acetobutylicum* using the CSH medium. During ABE fermentation, glucose is converted to pyruvate via the glycolysis pathway, and then, pyruvate is converted to butanol. Three key enzymes of glycolysis are glucokinase, phosphofructokinase and pyruvate kinase (PK). In this study, PK was up-regulated 22.34-fold with CaCO_3_ addition (Table 2). Another glycolysis pathway related enzyme 2, 3-bisphosphoglycerate-independent phosphoglycerate mutase (PGAM) which catalyzes the interconversion of 3- and 2-phosphoglycerate was also up-regulated 9.98-fold. The three key enzymes prior to acid and solvent production, namely, pyruvate flavodoxin/ferredoxin oxidoreductase (PFOR), pyruvate formate lyase (PFL) and acetyl CoA acetyltransferase (thiolase, THL) were up-regulated 4.26-fold, 5.93-fold and 23.08-fold, respectively. These enzymes were also involved in oxidation–reduction. In anaerobes, pyruvate is often oxidized to CO_2_ and acetyl-CoA by PFOR with the concomitant reduction of a low-potential redox protein, like ferredoxin (21.50-fold increase, Table 2) or flavodoxin. The enzyme responsible for this oxidative decarboxylation of pyruvate in many anaerobic bacteria is pyruvate flavodoxin/ferredoxin oxidoreductase (PFOR). The up-regulation of PFL in this study may respond to the presence of formic acid in the CSH medium though the reverse reaction from pyruvate to acetyl-CoA and formic acid by PFL is more common in the normal condition.

Ethanol and butanol were produced from acetyl-CoA and butyryl-CoA by the NADH dependent aldehyde/alcohol dehydrogenases (ADHE) and BDHs (BDHA and BDHB) with NADH consumption (Fig. 4—#3). The intracellular oxidation–reduction potential is reflected by the internal reducing equivalents (NADH/NAD^+^), which is important for the successful transformation from acid to solvent, and is regulated by the over-production of NADH^39^. The balance of NADH and ATP can be maintained by regulating acid production^19^. For example, two moles of NADH and four moles of ATP are generated through metabolizing one mole of glucose to two moles of acetate^19^.

Unexpectedly, ribulose-phosphate 3-epimerase, a key enzyme in the entry of hexose monophosphate (HMP) to glycolysis pathway was down-regulation with CaCO_3_ despite the increase in both glucose and xylose consumption (Fig. 2 and Table 2). Further study is required to understand the xylose consumption mechanism with the addition of CaCO_3_.

Since CSH contained microbial inhibitors and non-sugar impurities which could affect the quality of protein extraction, the number of proteins identified in this study was relatively low compared to those reported in previous studies^20,24^. Future study should be carried out to improve the quality of proteins from multipoint samples in order to fully illustrate the mechanism of enhanced butanol production from CSH by Ca^2+^ addition.

## Conclusion

This study further elucidated the effects of CaCO_3_ on direct ABE fermentation of pretreated corn straw biomass by *C. acetobutylicum* CICC8016. The results indicated that Ca^2+^ updated by *C. acetobutylicum* CICC8016 was inhibited by non-sugar biomass degradation products. Addition of calcium salts increased the intracellular levels of Ca^2+^, alleviating the inhibition on ABE production. Proteomics analysis revealed that most enzymes involved in glycolysis, redox balance and amino acid metabolism were up-regulated.

## Materials and methods

### Strain and seed culture

*Clostridium acetobutylicum* CICC 8016 used in this study was purchased from the CICC (China Center of Industrial Culture Collection, Beijing, China) and was preserved as a cell suspension in 30% (v/v) sterile glycerol at − 80 °C. Preculture medium (corn mash medium) was prepared by boiling 50 g of corn flour in 1 L of distilled water for 15–20 min and then autoclaved at 121 °C for 30 min. The strain stored at − 80 °C was firstly cultivated in 10 mL of the preculture medium at 37 °C for 7 days to induce sporulation. Afterwards, 1.0 mL of the spore culture was transferred to a test tube containing 9 mL of the same preculture medium and heat-shocked for 110 s at 100 °C to kill the non-sporulated bacteria, followed by water cooling to room temperature. Subsequently, the heat shocked spore suspension was activated at 38 °C for 48 h. To prepare seed culture, three test tubes (about 30 mL) were transferred to a 300 mL sealed conical bottle containing 270 mL fresh corn mash medium, and incubated statically at 38 °C for 24 h to achieve a cell density of 1.0 × 10^6^ cells/mL for inoculation^13^.

### Corn straw pretreatment and hydrolysis

Corn straw used in this study was collected from Maozhuang corn experimental field of Henan Agricultural University (Zhengzhou, China). The corn straw sample was ground and passed a sieve with an aperture of 2.0 mm. Ground corn straw was dried overnight at 105 °C prior to pretreatment. For pretreatment, 100 g of ground dry corn straw was mixed with 800 mL of 1% (w/v) dilute sulfuric acid, followed by pretreatment at 121 °C for 60 min in an autoclave. After pretreatment, the mixture was cooled to room temperature, and pH of the mixture was adjusted to 4.8 with 5 M NaOH. The mixture was then hydrolyzed by cellulase (CTec2, Novozymes, China) at a dosage of 15 FPU/g initial corn straw weight. Enzymatic hydrolysis was conducted at 50 °C for 48 h. After enzymatic hydrolysis, corn stover hydrolysate (liquid) was separated from the solid residue by centrifugation at 10,000*g* for 10 min.

### ABE fermentation

CSH obtained after enzymatic hydrolysis was adjusted to pH 7.0 with 5 M NaOH and supplemented with 1.0 g/L yeast extract and 0.05 M CaCO_3_ (5.00 g/L) or 0.05 M CaCl_2_ (5.55 g/L) before being autoclaved at 115 °C for 15 min. After being autoclaved, the sterile CSH solution (1 L) was mixed with each (10 mL) of the following filter-sterilized (0.22 μm) stock solutions to prepare the CSH medium for ABE production. The stock solutions included (1) a buffer solution: 50 g/L K_2_HPO_4_, 50 g/L KH_2_PO_4_, and 220 g/L g ammonium acetate; (2) a mineral solution: 1 g/L MnSO_4_·H_2_O, 20 g/L MgSO_4_·7H_2_O, 1 g/L NaCl, and 1 g/L FeSO_4_·7H_2_O; and (3) a vitamin solution: 0.1 g/L thiamin, 0.1 g/L para aminobenzoic acid, and 0.001 g/L biotin according to a previous publication^40^. CSH media without additional calcium salts and a synthetic medium (P2 medium) were used as controls. The P2 medium was prepared in a similar approach to CSH medium. Firstly, a synthetic solution containing 20 g/L glucose, 10 g/L xylose and 1 g/L yeast extract was autoclaved at 115 °C for 15 min. After being autoclaved, the synthetic solution (1 L) was supplemented with each (10 mL) of filter-sterilized (0.22 μm) stock solutions to prepare the P2 medium for ABE production.

In order to investigate the effect of intracellular Ca^2+^ concentration on ABE fermentation, BATPA-AM (intracellular Ca^2+^ chelator) was added into the P2 medium at different amounts and times during ABE fermentation. In one trial (treatment 1), 20 μM BATPA-AM was added at 12 h. In one trial (treatment 2), 20 μM BATPA-AM was added at 12 h, followed by adding 10 μM BATPA-AM at 24 h. In another trial (treatment 3), 20 μM BATPA-AM was added at 12 h, followed by adding 20 μM BATPA-AM at 24 h. A trial without BATPA-AM addition was carried out as a control. ABE production was monitored and compared.

ABE fermentation was conducted statically at 38 °C for 72–108 h in 100 mL sealed conical bottles containing 100 mL working volume (90 mL fermentation media and 10 ml seed solution) without pH control. Samples of 2.0 mL were withdrawn every 12 h during ABE fermentation and centrifuged at 4 °C at 300*g* for 10 min to remove impurities with large particles (undissolved CaCO_3_ and precipitates from straw hydrolysate). The rest solution was further centrifuged at 1,2000*g* for 10 min to bacterial cells at 4 °C. The supernatants were collected for the determination of sugars, acids and ABE by chromatography methods. The sediment cells were washed with 2 mL 0.01 M PBS (pH 7.4) and then suspended in the PBS solution. 1 mL of suspended cells was collected for determination of intracellular Ca^2+^ concentration. The rest cell solution was centrifuged to remove the PBS solution, followed by resuspension and staining in 0.5 mL 2% crystal violet for 3 min. The stained cells were centrifuged at 12, 000*g* for 10 min and the supernatant was discarded. The cells were washed with 1 mL 0.01 M PBS (pH = 7.4) and re-suspended in the PBS solution. Cell density was determined by a simple cell counting method using a microscope. Other 5 mL or 12 mL samples withdrew every 12 h were centrifuged at 4 °C at 10,000*g* for 10 min. Supernatants were collected for pH or extracellular Ca^2+^ measurement.

### Measurement of extracellular and intracellular Ca^2+^

Extracellular Ca^2+^ concentrations were determined by EDTA complexometric titration method. Firstly, 50 mL conical flask contained 10 mL supernatant was added with 1 mL 6 M HCl, mixed and boiled in a water bath for 30 s. After that, 5 mL 20% NaOH and 80 mg calcein-phenolphthalein mix indicator was added to the boiled solution and mixed well. The solution was titrated by 0.01 M EDTA standard solution and the volume of the titration solution was recorded when the solution color turned from yellowish-green to red. The Ca^2+^ concentration in the sample was calculated based on the consumed EDTA indicator volume.

The amounts of intracellular Ca^2+^ were determined by using a fluorescent Ca^2+^ indicator Fura-3-acetoxymethyl ester (Fura-3) (Solarbio, Beijing, China) as described previously^41,42^. Fura-3 was suspended in HBSS solution (Hank's Balanced Salt Solution, Solarbio) at a concentration of 5 mM, and then 200 μL of Fura-3 solution was used to load bacterial cells (collected form 1 mL sample) for 40 min at 37 °C under darkness, followed by washing the cells three times using 1 mL HEPES solution per wash. The washed cells were re-suspended at 1 × 10^5^ cells/mL in HEPES solution, immediately followed by a fluorescence assay. The fluorescence intensity was determined at 488 nm (excitation) and 526 nm (emission) using Cary Eclipse Fluor spectrophotometer (Varian, American). The intracellular Ca^2+^ concentration (based on 1 × 10^5^ cells/mL) was calculated according to the florescence intensity^41^.

### Protein extraction and proteomics analysis

To investigate the effect of CaCO_3_ on proteome, proteins were extracted from the 36 h samples (from fermentation with 50 mM CaCO_3_ and without calcium salt addition) in the denatured form using the following procedure. Fresh cells collected from the 12 mL sample were mixed with 200 μL denature buffer consisting of 1 mM EDTA, 1 mM PMSF (protease inhibitor) and 0.1 g protein extraction powder, followed by grinding with a mortar and pestle for 3 min. The mixture was centrifuged at 4 °C and 12,000*g* for 10 min. The protein in the supernatant was purified by the TCA-acetone method as follows^43^. Briefly, 2000 μg of each crude protein extract was resolved in the first dimension [isoelectric focusing (IEF)] inner diameter 3.3 mm containing 2% Ampholine pH range 4–7 (GE Healthcare, USA) using the following procedure in order: 50 V for 12 h, 200 V for 1 h, 1,000 V for 1 h, 9000 V for 2 h, 9000 V for 8 h, and finally 500 V for 12 h. The isoelectric focused gels were immersed in 2 mL 2D equilibration buffers with 1% dl-dithiothreitol (DTT), incubated for 15 min, followed by adding 2 mL 2D equilibration buffer with 2% iodoacetamide (IAA) and incubated for another 15 min. In the second dimension, proteins were separated and visualized on 18.5-cm-wide dried-slab gels by using Coomassie blue G-250 staining^44^. Tropomyosin (1 μg) was added to each protein sample as an internal standard for IEF prior to loading. Triplicate gels were run and analyzed for each protein sample. Gel images were captured with a Gel imaging and analysis system (Alpha Innotech, USA) and analyzed using PDQquest software (Version 8.0, Bio-Rad).

All spots of interest and all varying spots were outlined, quantified, and matched on all the gels. Spots of interest (n = 24, fold change > 1.5, *p* < 0.05) were sequenced by MALDI–TOF–MS/MS (5800 MALDI-TOF/TOF Analyzer, ABI). Mascot 2.2 software was used to identify the isoelectric point (pI), molecular weight (MW) of proteins followed by validation with EXPASY Bioinformatics Resource Portal (https://expasy.org).

### Chromatography analysis

ABE concentrations were measured by a gas chromatography (GC) system (Agilent 7890A GC, Agilent Technologies, USA) equipped with a flame ionization detector (FID) and an Agilent HP-FFAP capillary column (30 m length × 0. 320 mm internal diameter × 1 μm film thickness). Propanol was used as an internal standard. The oven temperature was initially maintained at 45 °C for 1 min, and then increased to 55 °C at a speed of 15 °C/min and maintained for 1 min, and then increased to 80 °C at a speed of 15 °C/min and maintained for 0.5 min, and then increased to 120 °C at a speed of 20 °C/min and maintained at 120 °C for 1 min. High purity nitrogen was used as a carrier gas at a speed of 1.5 mL/min. The temperatures of the injector and detector were maintained at 200 °C and 250 °C, respectively.

The concentrations of sugars, formic acid, acetic acid and butyric acid in the samples were measured by a high-performance liquid chromatography (HPLC) system (Dionex P680, Dionex, USA) equipped with an Aminex HPX-87H organic acid column (Bio-Rad, Hercules, CA) and a refractive index detector (Shodex RI-101, Showa Denko, Japan). The column temperature was operated at 55 °C with a detector temperature of 35 °C. The mobile phase was 5 mM H_2_SO_4_ at a flow rate of 0.60 mL/min.

The concentrations of furans, phenolic acids and phenolic aldehydes in the samples were measured by the other HPLC system (Waters 2695, Waters, USA) equipped with an ultraviolet detector (Waters 2489). The mobile phase consisted of acetic acid and methanol at a flow rate of 0.60 mL/min. The elution was conducted with 72% acetic acid for 0–15 min, followed by 53% acetic acid for 15–35 min, respectively.

## Supplementary information

Supplementary Figure S1.
