# Supplementary material for: Reassessment of the role of CaCO3 in n-butanol production from pretreated lignocellulosic biomass by Clostridium acetobutylicum
Source: Sci Rep. 2020 Oct 21;10:17956. doi: 10.1038/s41598-020-74899-9 (PMC7578090; doi:10.1038/s41598-020-74899-9)
Supplement: Supplementary file 1 — Supplementary Figure S1. [file 41598_2020_74899_MOESM1_ESM.pdf]

Reassessment of the Role of $CaCO_3$ in n-Butanol Production from Pretreated Lignocellulosic Biomass by *Clostridium acetobutylicum*

Zengping Su[1], Fengqin Wang[1*], Yaohuan Xie[1], Hui Xie[1], Guotao Mao[1], Hongsen Zhang[1], Andong Song[1*], Zhanying Zhang[2,3]

1. Key Laboratory of Agricultural Microbial Enzyme Engineering (Ministry of Agriculture), College of Life Science, Henan Agricultural University, Zhengzhou 450002, China

2. Centre for Agriculture and the Bioeconomy, Institute for Future Environments, Queensland University of Technology, Brisbane, Queensland 4000, Australia

3. School of Mechanical, Medical and Process Engineering, Science and Engineering Faculty, Queensland University of Technology, Brisbane, Queensland 4000, Australia

Corresponding authors:

*Fengqin Wang

w_fengqin@Henau.edu.cn

*Andong Song

songandong@henau.edu.cn

College of Life Science, Henan Agricultural University, No.63, Nongye Road, Jinshui District, Zhengzhou 450002, Henan Province, China.

Supplementary Material

Legend of supplementary Figure

Figure S1. ABE fermentation using P2 media with and without intracellular Ca^{2+} chelator BAPTA-AM. Control, no BAPTA-AM addition; Treatment 1, 20 μM BAPTA-AM at 12 h; Treatment 2, 20 μM BAPTA-AM at 12 h and 10 μM BAPTA-AM at 24 h; Treatment 3, 20 μM BAPTA-AM at 12 h and 20 μM BAPTA-AM at 24 h.

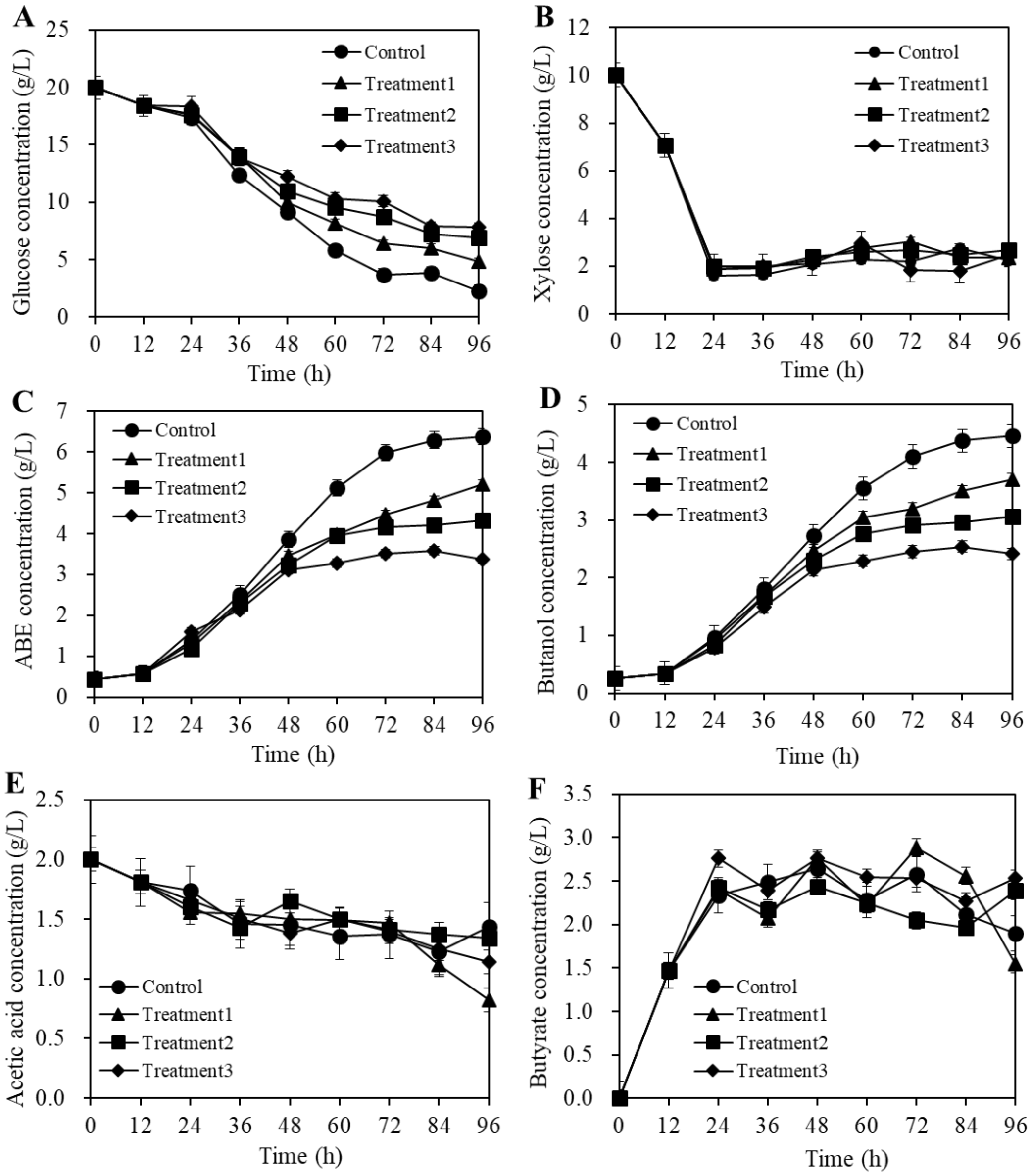
A
Glucose concentration (g/L)
Time (h)
B
Xylose concentration (g/L)
Time (h)
C
ABE concentration (g/L)
Time (h)
D
Butanol concentration (g/L)
Time (h)
E
Acetic acid concentration (g/L)
Time (h)
F
Butyrate concentration (g/L)
Time (h)
Control
Treatment1
Treatment2
Treatment3

Figure S1
